# Functionally active TRPA1 ion channel is downregulated in peptidergic neurons of the Edinger-Westphal nucleus upon acute alcohol exposure

**DOI:** 10.3389/fcell.2022.1046559

**Published:** 2023-01-10

**Authors:** Ammar Al-Omari, Miklós Kecskés, Balázs Gaszner, Tünde Biró-Sütő, Balázs Fazekas, Gergely Berta, Mónika Kuzma, Erika Pintér, Viktória Kormos

**Affiliations:** ^1^ Department of Pharmacology and Pharmacotherapy, Centre for Neuroscience, Szentágothai Research Centre, Medical School and Molecular Pharmacology Research Group, University of Pécs, Pécs, Hungary; ^2^ Medical School, Institute of Physiology, University of Pécs, Pécs, Hungary; ^3^ Department of Anatomy, Centre for Neuroscience, Medical School and Research Group for Mood Disorders, University of Pécs, Pécs, Hungary; ^4^ Department of Medical Biology, Medical School, University of Pécs, Pécs, Hungary; ^5^ Department of Forensic Medicine, Medical School, University of Pécs, Pécs, Hungary

**Keywords:** alcohol, centrally projecting Edinger-Westphal nucleus, transient receptor potential ankyrin 1, urocortin 1, cocaine-and amphetamine-regulated transcript, JT010

## Abstract

**Introduction:** The centrally projecting Edinger-Westphal nucleus (EWcp) contributes to the control of alcohol consumption by its urocortin 1 (UCN1) and cocaine- and amphetamine-regulated transcript (CART) co-expressing peptidergic neurons. Our group recently showed that the urocortinergic centrally projecting EWcp is the primary seat of central nervous system transient receptor potential ankyrin 1 (TRPA1) cation channel mRNA expression. Here, we hypothesized that alcohol and its metabolites, that pass through the blood-brain barrier, may influence the function of urocortinergic cells in centrally projecting EWcp by activating TRPA1 ion channels. We aimed to examine the functional activity of TRPA1 in centrally projecting EWcp and its possible role in a mouse model of acute alcohol exposure.

**Methods:** Electrophysiological measurements were performed on acute brain slices of C57BL/6J male mice containing the centrally projecting EWcp to prove the functional activity of TRPA1 using a selective, potent, covalent agonist JT010. Male TRPA1 knockout (KO) and wildtype (WT) mice were compared with each other in the morphological studies upon acute alcohol treatment. In both genotypes, half of the animals was treated intraperitoneally with 1 g/kg 6% ethanol vs. physiological saline-injected controls. Transcardial perfusion was performed 2 h after the treatment. In the centrally projecting EWcp area, FOS immunohistochemistry was performed to assess neuronal activation. TRPA1, CART, and urocortin 1 mRNA expression as well as urocortin 1 and CART peptide content was semi-quantified by RNAscope *in situ* hybridization combined with immunofluorescence.

**Results:** JT010 activated TRPA1 channels of the urocortinergic cells in acute brain slices. Alcohol treatment resulted in a significant FOS activation in both genotypes. Alcohol decreased the *Trpa1* mRNA expression in WT mice. The assessment of urocortin 1 peptide immunoreactivity revealed lower basal urocortin 1 in KO mice compared to WTs. The urocortin 1 peptide content was affected genotype-dependently by alcohol: the peptide content decreased in WTs while it increased in KO mice. Alcohol exposure influenced neither CART and urocortin 1 mRNA expression nor the centrally projecting EWcp/CART peptide content.

**Conclusion:** We proved the presence of functional TRPA1 receptors on urocortin 1 neurons of the centrally projecting EWcp. Decreased *Trpa1* mRNA expression upon acute alcohol treatment, associated with reduced neuronal urocortin 1 peptide content suggesting that this cation channel may contribute to the regulation of the urocortin 1 release.

## 1 Introduction

Alcohol use disorders are responsible for 3 million deaths worldwide each year, accounting for 5.3% of all deaths. The pathogenic role of alcohol exposure is known for more than 200 kinds of diseases (WHO 2018). Adverse consequences of addiction include mental and behavioral changes, mood disorders, and depression. Consequent social and economic damage, family and workplace conflicts highlight the importance of this research topic.

The midbrain Edinger-Westphal nucleus (EW) consists of two distinct cell populations. The preganglionic division provides cholinergic parasympathetic preganglionic fibers to the ciliary ganglion, to control pupil constriction and lens accommodation by the oculomotor nerve. The other division is peptidergic, designated as centrally projecting EW (EWcp) (for a review see Kozicz et al., 2011). Our research team has been investigating the role of the EWcp in stress adaptation response, mood control (Gaszner and Kozicz, 2003; Gaszner et al., 2004; Gaszner et al., 2007; Gaszner et al., 2012; Rouwette et al., 2011; Kormos and Gaszner, 2013; Kormos et al., 2022; Ujvári et al., 2022) and energy metabolism (Shah et al., 2013; Xu et al., 2014; Xu et al., 2022; Füredi et al., 2017) for many years.

The EWcp neurons express several reward-, stress- and energy expenditure-related neuropeptides, e.g., cholecystokinin, pituitary adenylate cyclase-activating polypeptide and substance P (Zuniga and Ryabinin, 2020; Priest et al., 2021), but the vast majority of peptidergic neurons co-express urocortin 1 (UCN1) and cocaine and amphetamine-regulated transcript peptide (CART) (Kozicz, 2003; Priest et al., 2021; Li and Ryabinin 2022). Numerous studies have provided evidence for the involvement of the latter two EWcp neuropeptides in the actions of alcohol and other drugs of abuse (Ong and McNally, 2020; Zuniga and Ryabinin, 2020).

UCN1 is a member of the corticotropin-releasing hormone (CRH) neuropeptide family. UCN1 binds both to the CRH1 and CRH2 receptors, and it shows higher affinity to the latter than CRH itself (Vaughan et al., 1995; Janssen and Kozicz, 2013). Interestingly, all efferent connections of the EWcp that project to addiction-related areas such as the ventral tegmental area (VTA), central nucleus of the amygdala (CeA), dorsal raphe nucleus (DRN), bed nucleus of the stria terminalis (BNST), lateral hypothalamus (LH) and lateral septum (LS) are urocortinergic (Zuniga and Ryabinin, 2020) and importantly, these areas were shown to express CRH receptors (Schreiber and Gilpin 2018). Multiple genetic studies have demonstrated that high alcohol preference in mouse and rat strains was associated with increased UCN1 levels (Bachtell et al., 2002; Bachtell et al., 2003; Turek et al., 2005; Fonareva et al., 2009; Ryabinin et al., 2012). Lesions of the rodent EW greatly attenuate ethanol preference (Bachtell et al., 2004; Ryabinin and Weitemier, 2006). UCN1 cells display a robust FOS (a marker of acute neuronal activity) response upon exposure to both passive and self-administered ethanol (Bachtell et al., 1999; Ryabinin et al., 2001; Weitemier et al., 2001; Zuniga and Ryabinin, 2020). The amount of consumed ethanol correlates positively with the number of FOS positive cells and *Fos* mRNA expression in the EWcp (Sharpe et al., 2005; Giardino et al., 2017). Additionally, increased level of FOSB (a marker of chronic neuronal activity) was observed in the EWcp after a seven-day-period of 24 h access to ethanol in mice (Bachtell et al., 1999; Ozburn et al., 2012).

CART is a neuropeptide, implicated in energy metabolism (Kristensen et al., 1998; Farzi, et al., 2018; Lau et al., 2018) and regulation of feeding, drug reward and addictive behaviors (Vicentic and Jones, 2007; Zuniga and Ryabinin 2020; Ong and McNally, 2020). CART is expressed in appetite, motivation and reward-related brain areas (e.g., EWcp, paraventricular nucleus of the hypothalamus, arcuate nucleus, dorsomedial hypothalamus, LH, nucleus accumbens, amygdala, locus coeruleus, nucleus of the solitary tract, medial accessory olive) (Koylu et al., 1998; Elias et al., 2001). Although several studies were published investigating the importance of CART in addiction, only few studies are available on the role of EWcp/CART in alcohol consumption. For instance, low alcohol preference DBA/2J mice show reduced CART expression at mRNA and peptide level in the EWcp compared to alcohol preferring C57BL/6J mice (Giardino et al., 2017). Moreover, reduced alcohol intake and preference was observed in *Cart* KO mice compared to the WTs in a 24 h 2-bottle-choice procedure (Salinas et al., 2014).

The fact that UCN1 and CART fully co-localize in the EWcp, and they show increased peptide and mRNA levels in alcohol preferring mice, suggests their important and probably common role in the regulation of alcohol intake and related behaviors.

The transient receptor potential ankyrin 1 (TRPA1) is a non-selective cation channel. The role of the peripheral TRPA1 in nociception and inflammatory responses has been well established (Kádková et al., 2017; Jannis et al., 2019; Talavera et al., 2020), in contrast, only limited knowledge has accumulated on its role in the central nervous system. In our recent studies, we proved that urocortinergic neurons in EWcp uniquely express significant amount of *Trpa1* mRNA in the mouse (Kormos et al., 2022). However, an earlier calcium imaging study described the link between ethanol and TRPA1, it was examined exclusively in the context of pain: ethanol activated the human TRPA1 on human embryonic kidney-derived 293 (HEK293) cells (Komatsu et al., 2012). Alcohol is metabolized by alcohol dehydrogenase (ADH) into the reactive and toxic intermediate product acetaldehyde, which is rapidly converted into acetic acid (Cederbaum, 2012). Acetaldehyde is considered as the major contributor of the detrimental effects by acute and chronic alcohol consumption including flushing, headache, cirrhosis, and cancer (Eriksson, 2001). Human and mouse TRPA1 receptors were activated specifically by acetaldehyde both in a HEK293T cell heterologous expression system and cultured mouse trigeminal neurons and the pharmacological inhibition of the TRPA1 receptor prevented the acetaldehyde-induced activation (Bang et al., 2007). Acetic acid was also shown to acitivate TRPA1 in trigeminal neurons in patch clamp recordings and Ca^2+^ microfluorometry (Wang et al., 2011).

Since, we showed recently the presence of the *Trpa1* only at mRNA level in urocortinergic neurons (Kormos et al., 2022) in this study we aimed to investigate whether the ion channel is functionally active in the EWcp neurons. Considering the involvement of UCN1 in acute and chronic alcohol consumption (Schreiber and Gilpin, 2018; Zuniga and Ryabinin, 2020), in this study we aimed to test if TRPA1 contributes to the recruitment of EWcp/UCN1/CART neurons in acute alcohol exposure.

## 2 Materials and methods

### 2.1 Animals

Animals were housed in a temperature and humidity controlled 12 h light-dark cycle environment (lights on at 6 a.m.) in standard polycarbonate cages (365 mm × 207 mm × 144 mm) in four to six mice per cage groups, at the animal facility of the Department of Pharmacology and Pharmacotherapy, University of Pécs. Mice were provided *ad libitum* with standard rodent chow and tap water. All procedures were approved by the Animal Welfare Committee at Pécs University, National Scientific Ethical Committee on Animal Experimentation in Hungary (BA02/2000-25/2021) in agreement with the directive of the European Communities Council in 1986, and with the Law of XXCIII, in 1998, on Animal Care and Use in Hungary.

The original breeding pairs of *Trpa1* KO mice were obtained from Prof. P. Geppetti, University of Florence, Italy. *Trpa1* KO mice were bred on C57BL/6J background and crossed back after 10 generations (Kormos et al., 2022). WT and KO mice were selected from different litters. Offspring were genotyped for *Trpa1* gene by PCR (sequences of primers: ASM2: ATC ACC TAC CAG TAA GTT CAT; ASP2: AGC TGC ATG TGT GAA TTA AAT).

### 2.2 Experimental design

Acute coronal brain slices containing the EWcp from 4 to 5 week-old male C57BL/6J mice (*n* = 10) were used for the electrophysiological recordings to prove the presence of functional TRPA1 channel using the potent and selective agonist JT010.

In an independent experiment, 9–12 week-old male *Trpa1* knockout (KO) mice and their wildtype (WT) counterparts were assigned to four experimental groups: *Trpa1* KO (*n* = 7) and WT (*n* = 7) mice were intraperitoneally (i.p.) injected with 6% ethanol (D = 1 g/kg), while another set of *Trpa1* KO (*n* = 8) and WT (*n* = 6) mice were injected with the same volume of physiological saline as a control (Korkosz et al., 2006; Pradhan et al., 2013). Mice were euthanized 2 h after the treatment for the morphological studies, which is the time required after the onset of the stimuli to reach the peak of FOS protein expression (Chaudhuri et al., 2000; Zhong et al., 2014).

### 2.3 Acute brain slice preparation for electrophysiology

Electrophysiology experiments were performed in acute coronal brain slices containing the EWcp (from Bregma −2.92 to −4.04 according to Paxinos and Franklin, 2001) taken from C57BL/6J mice. Under deep isoflurane anesthesia, mice were decapitated and 300 µm thick coronal slices were cut in ice-cold external solution containing (in mM): 93 NMDG, 2.5 KCl, 25 Glucose, 20 HEPES, 1.2 NaH_2_PO_4_, 10 MgSO_4_, .5 CaCl_2_, 30 NaHCO_3_, 5 L-ascorbate, 3 Na-pyruvate, 2 thiourea bubbled with 95% O_2_ and 5% CO_2_. Slices were transferred to artificial cerebrospinal fluid (ACSF) containing (in mM) 2.5 KCl, 10 glucose, 126 NaCl, 1.25 NaH_2_PO_4_, 2 MgCl_2_, 2 CaCl_2_, 26 NaHCO_3_ bubbled with 95% O_2_ and 5% CO_2_. After an incubation period of 10 min at 34°C in the first solution, the slices were maintained at room temperature in ACSF until use. After recordings, the sections were immersed into fixative (4% paraformaldehyde in 0.1 M PB) for overnight fixation, then 50 µm thick coronal slices were re-sectioned using a Leica VT1000S vibratome (Leica Biosystems, Wetzlar, Germany) for further immunostaining.

### 2.4 *In vitro* electrophysiological recordings

Patch pipettes were pulled from borosilicate glass capillaries with filament (1.5 mm outer diameter and 1.1 mm inner diameter; Sutter Instruments, Novato, CA, United States) with a resistance of 2–3 MΩ. The pipette recording solution contained (in mM) 3.5 KCl, 40 CsCl, 90 K-gluconate, 1.8 NaCl, 1.7 MgCl_2_, 0.5 EGTA, 10 Hepes, 2 Mg-ATP and .2% Biocytin, pH 7.3 adjusted with KOH; 290–300 mOsm. Whole-cell recordings were made with Axopatch 700B amplifier (Molecular Devices, San José, CA, United States) using an upright microscope (Eclipse FN1, Nikon) with 40× (NA: .8) water immersion objective lens equipped with differential interference contrast (DIC) optics. DIC images were captured with an Andor Zyla 5.5 s CMOS camera (Oxford Instruments, Abingdon, United Kingdom). All recordings were performed at 32°C, in ACSF bubbled with 95% O_2_ and 5% CO_2_. Cells with lower than 20 MΩ access resistance (continuously monitored) were accepted for analysis. Signals were low-pass filtered at 5 kHz and digitized at 20 kHz (Digidata 1550B, Molecular Devices). When it is indicated 5 μM JT010, 10 µM CNQX (Sigma) and 1 µM Gabazine (Sigma) were applied to the bath solution. In these experiments membrane potential was manually adjusted (max. −50 pA) to keep the neuron just below the threshold for action potential (AP) firing. This method allowed us the easily monitor the effect of TRPA1 activation since ∼5 mV depolarization already induced AP firing.

### 2.5 Perfusion and tissue collection

WT and *Trpa1* KO mice were euthanized 2 h after the alcohol administration with an overdose of urethane (2.4 g/kg) injected intraperitoneally. Then, tail clipping was performed to validate their genotype, and urine was collected by a urinary bladder puncture into a syringe. The samples were filled into pre-chilled tubes and stored at −20°C for urine alcohol concentration (UAC) measurements. Then, mice were perfused transcardially by 20 mL of ice-cold 0.1 M phosphate-buffered saline (PBS) (pH: 7.4) followed by 150 mL 4% paraformaldehyde (PFA) solution in Millonig buffer (pH 7.4).

Brain samples were dissected and post-fixed for 72 h at 4°C in PFA solution. The brains were coronally sectioned using a Leica VT1000S vibratome (Leica Biosystems, Wetzlar, Germany). Four series of 30 μm sections were collected and stored in PBS containing sodium-azide (.01%) at 4°C, and for long term storage at −20°C, they were transferred into antifreeze solution. Four representative sections of the EWcp (from Bregma −2.92 to −4.04 according to Paxinos and Franklin, 2001) per animal were selected for each staining.

### 2.6 Urine alcohol concentration measurement

The ethanol content of the urine samples was examined by headspace gas chromatography with flame-ionization detection (Agilent 7890A GC system, G1888 Network Headspace Sampler). 50 µL of sample was added to 500 µL of internal standard solution (tert-butanol solution with a concentration of 0.05 g/L) previously introduced into a 20-mL headspace vial. The vial was crimp sealed and thermostated at 75°C ± 0.1°C. After the equilibrium was established (15 min), 2 µL of vapor was injected directly into the chromatographic columns (DB-ALC1, Agilent J&W Scientific, 30 m × 0.32-mm i.d., 1.8-µm film thickness and DB-ALC2, Agilent J&W Scientific, 30 m × .32-mm i.d., 1.2-µm film thickness). The HS loop and transfer line temperatures were set at 75°C and 85°C, respectively. The injection port temperature was held at 150°C and used in split mode with a split ratio of 5:1. The flame ionization detector (FID) temperature was maintained at 260°C. Nitrogen was used as carrier gas. The GC oven temperature was kept at 35°C during the run time (4 min). The analytical method was validated for system suitability, selectivity, accuracy, linearity, repeatability, and intermediate precision in accordance with the current ICH guidelines [https://www.ema.europa.eu/en/documents/scientific-guideline/ich-guideline-q2r2-validation-analytical-procedures-step-2b_en.pdf (accessed 17 August 2022)]. Detector response was linear over the range of 0.025–2.5 g/L for both acetaldehyde and ethanol. The detection limit (DL) and quantitation limit (QL) values of both compounds were found to be 0.015 g/L and 0.025 g/L, respectively.

### 2.7 RNAscope *in situ* hybridization combined with immunofluorescence

RNAscope *in situ* hybridization (ISH) was performed to measure the expression of *Trpa1*, *Cart,* and *Ucn1* mRNA in EWcp. The pretreatment procedure was optimized for 30 µm-thick PFA-fixed sections (Kormos et al., 2022). Further steps of RNAscope (probe hybridization, signal amplification and channel development) were performed according to RNAscope Multiplex Fluorescent Reagent Kit v2 user manual (ACD, Hayward, CA, United States). Mouse *Trpa1* (ACD; Cat. No.: 400211), *Cart* (ACD; Cat. No.: 432001) and *Ucn1* probes (ACD; Cat. No.: 466261) were visualized by cyanine 3 (Cy3) (1:750 for *Trpa1* and 1:3000 for *Cart*) and fluorescein (1:3000 for *Ucn1*) dyes, respectively.

In case of the *Trpa1,* ISH was combined with immunofluorescence for UCN1 to examine the peptide content also. After the RNAscope procedure, slides were treated with polyclonal rabbit anti-UCN1 antibody (RRID: AB 2315527, gift from Prof. Wylie W. Vale, Salk Institute La Jolla, CA, United States) diluted to 1:20.000, for 24 h at 24°C. After 2 min × 15 min washes, Alexa 488-conjugated donkey anti-rabbit antibody (Jackson Immunoresearch Europe Ltd., Cambridgeshire, United Kingdom; Cat. No: 711-545-152, diluted to 1:500) was used for 3 h at 24°C. Sections were counterstained with DAPI (ACD) and covered with ProLong Gold Antifade (Thermo Fisher Scientific, Waltham, MA, United States) mounting medium.

Mouse 3-plex positive (ACD; Cat. No: 320881) control probes specific to *Polr2a* mRNA (fluorescein), *Ppib* mRNA (Cy3) and *Ubc* mRNA (cyanine 5, Cy5) and 3-plex negative (ACD; Cat. No: 320871) control probes to bacterial *dabP* mRNA were tested on the EWcp. The 3-plex positive control probes gave well-detectable signal in the EWcp, while the negative control probes did not give any recognizable fluorescence in the preparations (images not shown).

The specificity of the rabbit UCN1 (RRID: AB 2315527, gift from WW Vale, Salk Institute La Jolla, CA, United States) was tested earlier in mice (see our earlier work Kormos et al., 2016). In this study, omission or replacement of primary and secondary antibodies by non-immune sera abolished labeling in both WT and KO mice (images not shown).

### 2.8 Immunohistochemistry with diaminobenzidine

FOS immunohistochemistry was performed to assess the acute neuronal activity in EWcp. Sections were washed three times in PBS and treated with 1% H_2_O_2_ (Sigma Chemical, Zwijndrecht, Netherlands) to quench endogenous peroxidase activity of the tissue. After 3 min × 10 min washes, sections were treated with .5% Triton X-100 (Sigma Chemical, Zwijndrecht, Netherlands) in PBS to enhance the permeability. Then, non-specific binding sites were blocked using 2% normal goat serum in PBS. Sections were then incubated with rabbit anti-cFOS polyclonal antibody (1:2.000, RRID: AB, 2231974 Synaptic Systems GmbH, Cat. No: 226 003) in a dilution overnight at room temperature. After 3 min × 10 min washes with PBS, sections were incubated with biotinylated anti-rabbit gamma globulin for 1 h (VECTASTAIN^®^ Elite ABC-HRP Kit, Peroxidase Rabbit IgG Vector Laboratories Cat. No: PK-6101). After washes again 3 times with PBS, sections were incubated in ABC (avidin-biotin complex) solution for 1 h. After washes they were treated with .05% diaminobenzidine (DAB) in Tris buffer with .003w/v% H_2_O_2_ (Sigma Chemical, Zwijndrecht, Netherlands), the latter reaction was controlled under a stereomicroscope and stopped with PBS. Sections were mounted on gelatin-coated glass slides, air-dried, treated with xylene (Merck, Leicester, United Kingdom) and coverslipped with DPX mounting medium (Merck, Leicester, United Kingdom).

The specificity of the FOS serum was tested in our recent work (Kovács et al., 2019) by preabsorption, using the respective blocking peptide (Synaptic Systems, Cat. No: 226-0P). Omission and replacement controls were performed also on some randomly selected sections collected in alcohol-treated mice, and no immunosignal was recognizable.

### 2.9 Immunofluorescence

In case of acute EWcp slices, Biocytin and UCN1 fluorescent labeling were performed to identify the electrophysiologically examined urocortinergic neurons. Sections were washed 2 min × 15 min with PBS then treated with .5% Triton X-100 (Sigma Chemical, Zwijndrecht, Netherlands) in PBS with Alexa 488-conjugated Streptavidin (Cat. No: 016-540-084 Jackson Immunoresearch Europe Ltd., Cambridgeshire, United Kingdom) diluted to 1:2.000, for 2 h. After washes, polyclonal rabbit anti-UCN1 antibody (RRID: AB 2315527, gift from Prof. Wylie W. Vale, Salk Institute La Jolla, CA, United States) was used diluted to 1:5.000, overnight at 24°C. After washes, Cy3-conjugated donkey anti-rabbit antibody (Cat. No: 711-165-152, Jackson) was used diluted to 1:500 for 3 h. Sections were mounted on gelatin-coated glass slides then air-dried and coverslipped with glycerol-PBS (1:1).

In case of acute alcohol exposure model, CART immunofluorescence was performed to semi-quantify the peptide content of the EWcp neurons. Sections were washed 2 min × 15 min with PBS then treated with .5% Triton X-100 (Sigma Chemical, Zwijndrecht, Netherlands) in PBS for 30 min and blocked with 2% normal donkey serum in PBS. Sections were incubated with anti-CART rabbit antibody [Phoenix H-003-62 (55–102)] in 1: 10.000 dilution overnight at room temperature. After 2 min × 15 min washes with PBS, sections were incubated with Cy3-conjugated donkey anti-rabbit antibody (Cat. No.: 711-165-152 Jackson) in 1: 500 dilution for 3 h. After washes, sections were mounted on gelatin-coated glass slides, air-dried and coverslipped with glycerol-PBS (1:1).

The specificity of the rabbit CART antibody (RRID: AB 2313614 Phoenix Europe GmbH, Karlsruhe, Germany) was tested earlier (Armbruszt et al., 2015). In this study, omission or replacement of primary and secondary antibodies by non-immune sera abolished labeling in both WT and *Trpa1* KO mice (images not shown).

### 2.10 Microscopy, digital imaging and morphometry

The DAB-labeled sections were studied and digitalized by a Nikon Microphot FXA microscope with a Spot RT camera (Nikon, Tokyo, Japan). The number of FOS-positive nuclei was determined by manual cell counting on the whole cross section surface area of the EWcp.

Fluorescent labeled sections were digitalized by an Olympus FluoView 1000 confocal microscope (Olympus, Europa, Hamburg, Germany) in sequential scanning in analogue mode. We used 80 µm confocal aperture (optical thickness 3.5 µm), 1024 × 1024-pixel resolution, and a ×60 objective for scanning. The excitation and emission spectra for the respective fluorophores were selected using built-in settings of the FluoView software (FV10-ASW; Version 0102, Olympus, Europa, Hamburg, Germany). DAPI was excited at 405 nm, Fluorescein and Alexa 488 at 488 nm and Cy3 at 550 nm. Sections were scanned for the respective wavelengths at three channels. Digital images of the three channels, depicting the same area, were automatically superimposed and merged. Co-localization was assessed on digital images showing virtual blue (DAPI), green (fluorescein), and red (Cy3) colors representing the fluorescent signals of the three channels.

The UCN1 and CART signals showed a confluent or cluster-like patterns both at mRNA and peptide level. As counting of individual fluorescent dots was not possible, the intensity of the fluorescence was measured by ImageJ software (version 1.42., NIH, Bethesda, MD) in 5–10 cell bodies using four non-edited images of the corresponding channel. The region of interest was manually determined at cytoplasmic areas of neurons. The signal density was corrected for the background signal. The average of the specific signal density (SSD) of 5–10 neurons was determined in four sections per animal. The average of these four values represented the SSD value of one mouse. The SSD was expressed in arbitrary units (a.u.).

The *Trpa1* mRNA signal appeared as well distinguishable scattered fluorescent dots. The number of dots per cell was manually counted in the 5-10 *Trpa1*-expressing neurons, in four sections per animal. Finally, these values were averaged as described above.

### 2.11 Statistics

Data were expressed as mean ± standard error of the mean for each experimental group. Data sets were tested for normality (Shapiro–Wilk test; Shapiro and Wilk, 1965) and homogeneity (Bartlett’s Chi-square test; Snedecor and Cochran, 1989) of variance. Outlier data beyond the two-sigma range were excluded. Data were evaluated by two-way analysis of variance (ANOVA). Tukey’s *post hoc* tests were performed based on first or second order effects in ANOVA tests.

Student’s *t*-test for independent samples was used to compare the *Trpa1* mRNA expression of alcohol treated vs. control WT mice. Analyses were conducted using Statistica 8.0 (StatSoft, Tulsa, OK) (alpha = 5%).

All statistical analyses of electrophysiological data were performed using Clampfit v. 10.7 (Molecular Devices) and OriginPro v. 8.6. Data were evaluated by Tukey’s *post hoc* test upon one-way ANOVA.

## 3 Results

### 3.1 Electrophysiology

**FIGURE 1:**
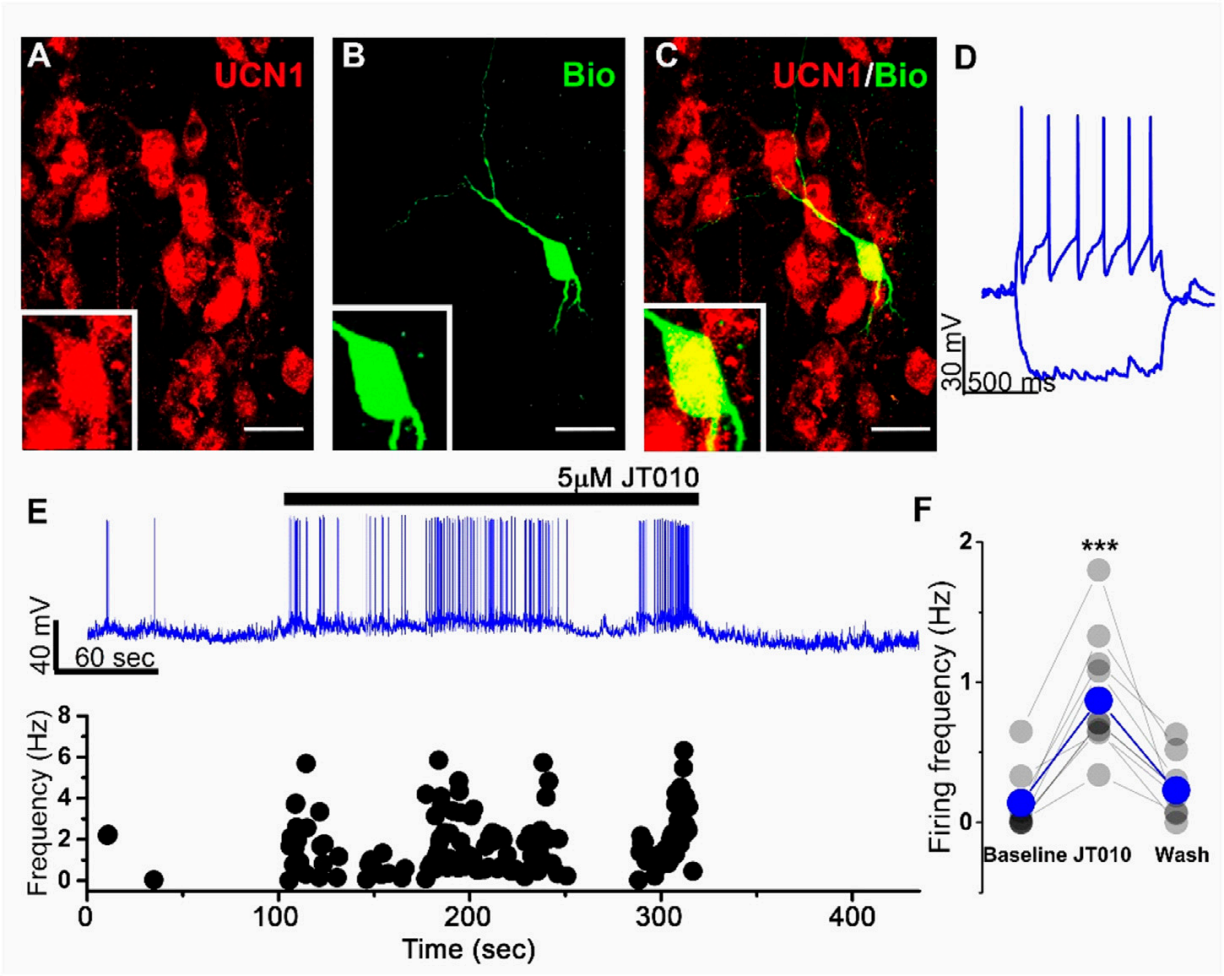
JT010 increases spontaneous firing frequency of UCN1-immunoreactive neurons in the EWcp nucleus. Representative confocal images of UCN1-immunoreactive (red) cells **(A)** and a biocytin (green) filled patched neuron **(B)** and the merged image **(C)**. Insets shows the magnified soma of the patched neuron. Scale bars: 40 µm. Response of the recorded cell **(D)** to 1 s current injection (−100 and +100 pA). Representative current clamp recordings (E, upper panel) showing the spontaneous activity of UCN1-immunoreactive neuron. Black bar represents JT010 application (5 µM). Instantaneous firing frequency **(E)**, lower panel] of each action potential in the upper recording is plotted. Statistics **(F)** showing the firing frequency at baseline (2 min before drug application) during JT010 application and after washing out the drug (*n* = 9 from 4 mice). ***p < .001; Tukey’s *post hoc* test upon one-way ANOVA.

To test whether TRPA1 is functionally active on EWcp neurons we performed slice patch clamp recordings in whole cell configuration. All patched neurons were filled with biocytin and tested for UCN1 immunopositivity *post hoc* Figures 1A–C. Only UCN1-immunoreactive cells were used in statistical analysis. UCN1-immunoreactive neurons were tonically active at resting membrane potential Figure 1D as it was shown previously (Topilko et al., 2022). We used JT010, a potent and selective, covalently binding agonist to activate TRPA1 (Takaya et al., 2015). Since recording of a relatively small transmembrane current in neurons can be challenging, we decided to monitor membrane potential changes in current clamp mode upon application of JT010. Resting membrane potential of recorded cells were adjusted via the amplifier to keep the cells just below the threshold for AP firing (few AP still occurred). This strategy prevented spontaneous firing, however even a moderate membrane potential depolarization -by the activation of TRPA1- resulted in high frequency firing. Firing frequency was significantly increased during JT010 application in UCN1-immunoreactive neurons (Figures 1E, F). AP frequency was .14 ± .07 Hz at baseline, .87 ± .17 Hz during drug application and .22 ± .07 Hz after washing out the drug (*n* = 9/4 mice) (Figure 1F). Interestingly, after bath application of fast synaptic blockers (CNQX and Gabazine) the effect of JT010 was still present indicating that it acts directly on UCN1 positive neurons (Supplementary Figure S2). Notably UCN1-immunonegative neurons in the EW region showed no change in firing frequency or in membrane potential upon the application of JT010 (Supplementary Figure S1).

### 3.2 UAC measurement

**FIGURE 2:**
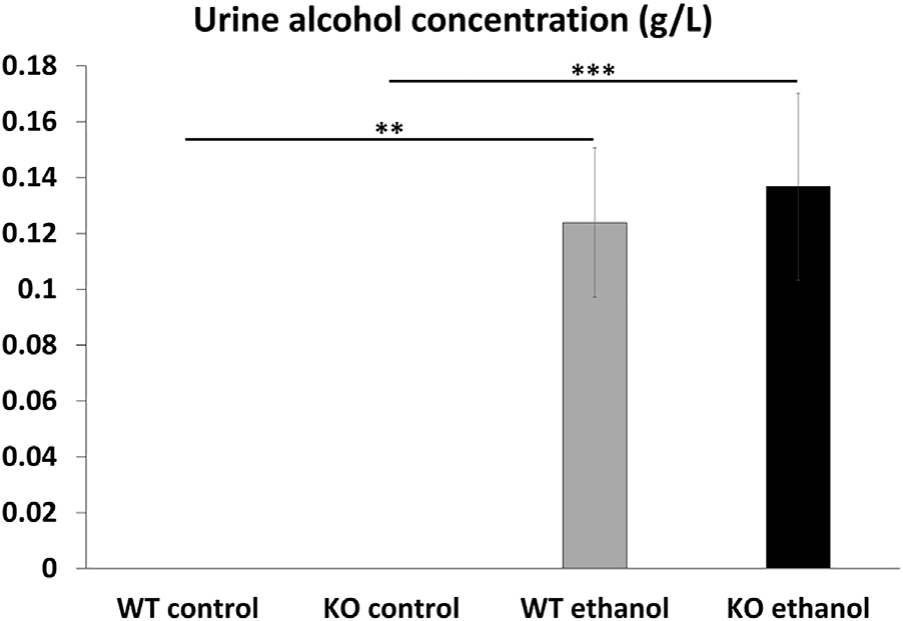
Urine alcohol concentration. Quantitative evaluation of alcohol concentration in the urine of wildtype (WT) and *Trpa1* knockout (KO) mice, 2 h after i.p., saline (control) or 1 g/kg ethanol injection. Columns show means ± SEM of alcohol concentration (g/L) (*n* = 6–8; **p = .002; ***p = .0009; Tukey’s *post hoc* test upon two-way ANOVA).

The ethanol content of the urine samples was examined by headspace gas chromatography. As expected, no ethanol was detectable in the urine of the saline-treated groups. We detected similar ethanol concentration in the urine of both alcohol-treated WT and *Trpa1* KO animals (ANOVA, main effect of treatment: F_1,24_ = 37.030; *p* < 10^−6^; main effect of genotype F_1,24_ = .09; *p* = .76) (Figure 2).

### 3.3 FOS immunohistochemistry

**FIGURE 3:**
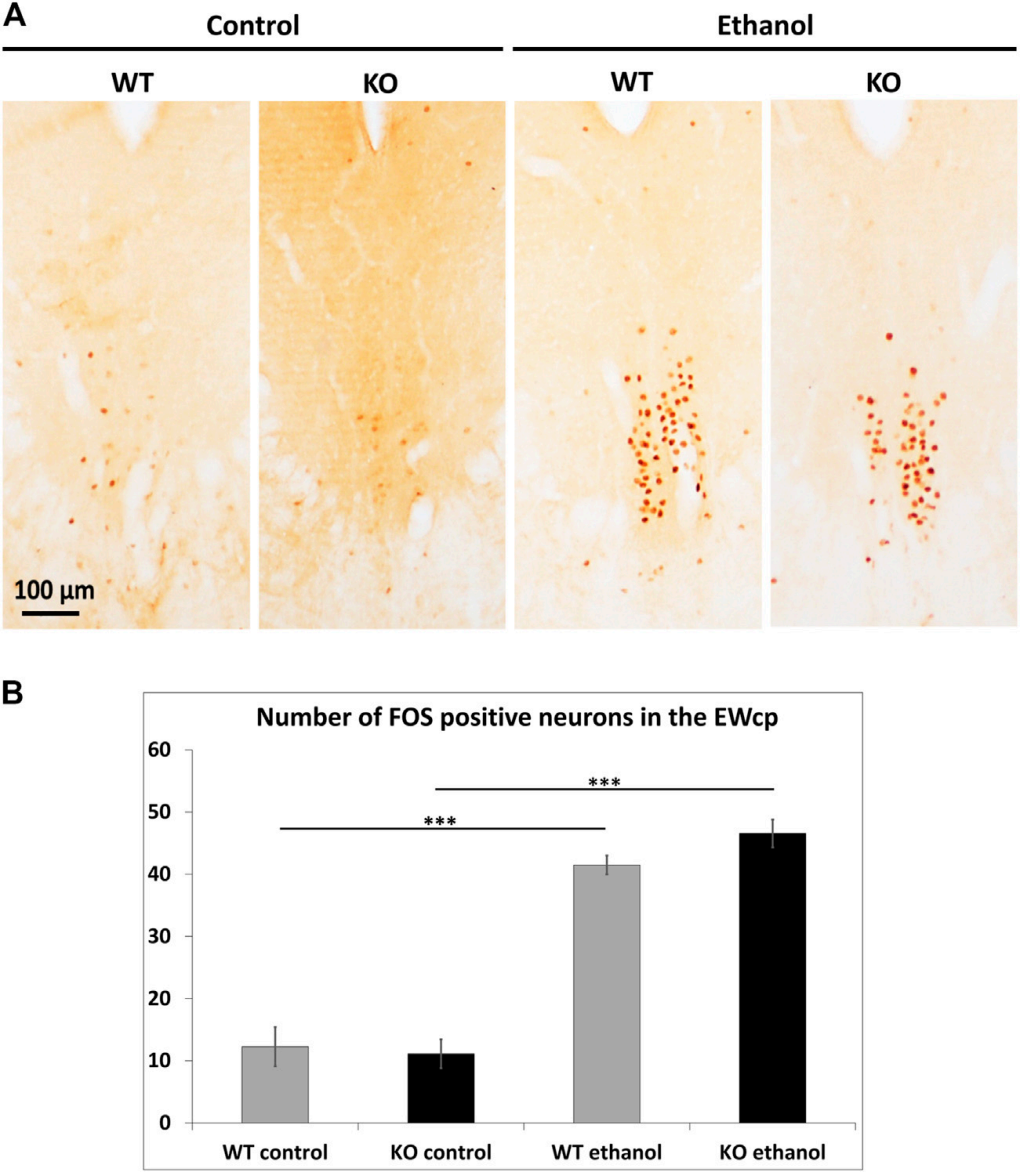
Quantitation of FOS immunoreactivity in the centrally projecting Edinger–Westphal nucleus (EWcp). **(A)** Representative immunohistochemical images showing the expression of FOS, as a marker of early neural activation, in the EWcp of wildtype (WT) and *Trpa1* knockout (KO) mice 2 h after i.p., saline (control) and 1 g/kg ethanol injection. Neuronal activation is represented by brown colored nuclei. **(B)** Quantitative evaluation of FOS immunostaining in the EWcp of WT and *Trpa1* KO mice, 2 h after i.p., saline (control) and 1 g/kg ethanol injection. Columns show means ± SEM of FOS positive neurons in the EWcp (*n* = 6–8; ***p = .0001; Tukey’s *post hoc* test upon two-way ANOVA).

FOS immunohistochemistry was performed to assess the acute neuronal activity in EWcp. Alcohol treatment resulted in an approximately 4-fold rise in the number of EWcp/FOS positive neurons in both WT and *Trpa1* KO animals compared to the respective controls (ANOVA, main effect of treatment: F_1,25_ = 183.33; *p* < 10^−6^) without the main effect of genotype (Figures 3A, B).

### 3.4 *Trpa1* RNAscope *in situ* hybridization

**FIGURE 4:**
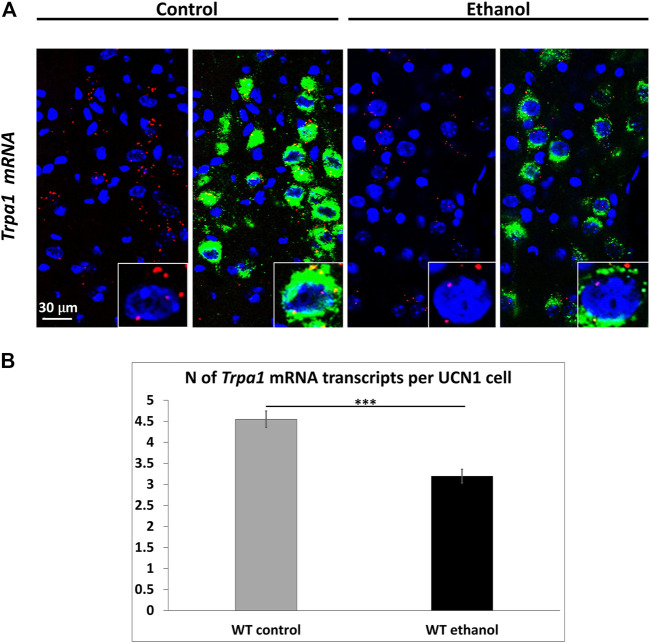
*Trpa1* mRNA expression in the centrally projecting Edinger–Westphal nucleus (EWcp) of control and ethanol-treated mice. **(A)** Representative fluorescence images showing the expression of *Trpa1* mRNA (red) by RNAscope *in situ* hybridization and its co-localization with the urocortin1 (UCN1) peptide (green) by immunofluorescence, in the EWcp of *Trpa1* wildtype (WT) mice 2 h after i.p., saline (control) and 1 g/kg ethanol injection. For nuclei, the sections were counterstained with 4′,6-diamidino-2-phenylindole (DAPI) (blue). **(B)** Quantitative evaluation of *Trpa1* mRNA expression in the EWcp of WT mice, 2 h after i.p., saline (control) and 1 g/kg ethanol injection. Columns show means ± SEM of *Trpa1* mRNA transcripts in the EWcp (*n* = 6–8; ***p = .0001; Student’s t-test).

RNAscope ISH was performed to assess the effect of alcohol on the number of *Trpa1* mRNA transcripts in the EWcp/UCN1 neurons of WT animals. *Trpa1* mRNA showed a full co-localization with the UCN1 peptide immunosignal in the EWcp (Figure 4A). Alcohol-treated mice showed a significantly lower number of *Trpa1* transcripts compared to the controls (t_12_ = 5.345; *p* = .0001) (Figure 4B).

### 3.5 Dynamics of UCN1 mRNA and peptide upon alcohol treatment

To examine the effect of alcohol on *Ucn1* mRNA expression and UCN1 peptide content in the EWcp neurons, RNAscope ISH and immunofluorescence were performed, respectively.

**FIGURE 5:**
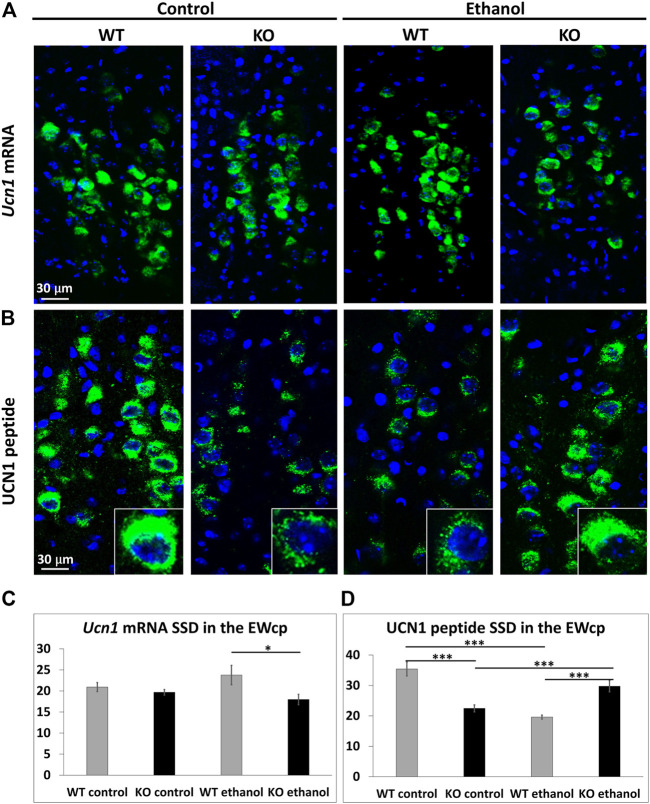
UCN1 mRNA and peptide content of the centrally projecting Edinger–Westphal nucleus (EWcp). Representative fluorescence images showing the expression of urocortin1 (*Ucn1*) mRNA (green) by RNAscope *in situ* hybridization **(A)** and the UCN1 peptide (green) by immunofluorescence **(B)**, in the EWcp of wildtype (WT) and *Trpa1* knockout (KO) mice 2 h after i.p., saline (control) and 1 g/kg ethanol injection. For nuclei, the sections were counterstained with 4′,6-diamidino-2-phenylindole (DAPI) (blue). Quantitative evaluation of *Ucn1* mRNA **(C)** and UCN1 peptide **(D)** specific signal density (SSD) in the EWcp of *Trpa1* WT and *Trpa1* KO mice, 2 h after i.p., saline (control) and 1 g/kg ethanol injection. Columns show means ± SEM of *Ucn1* mRNA **(C)** and UCN1 peptide **(D)** SSD in the EWcp (*n* = 6–8; *p = .03; ***p = .0001; two-way ANOVA and Tukey’s *post hoc* test).

There was a main effect of the genotype (ANOVA: F_1,23_ = 6.758; *p* = .016) on *Ucn1* mRNA expression. In control groups, no difference was detected, however a lower *Ucn1* mRNA expression was observed in KO animals upon alcohol treatment (*p* = .030) (Figures 5A, C).

A strong effect of genotype × treatment interaction was observed (ANOVA: F_1,22_ = 51.816; *p* < 10^−6^) on UCN1 at peptide level. The basal UCN1 content of the EWcp neurons was significantly lower in *Trpa1* KO mice compared to WTs (*p* < 10^−4^). Moreover, the UCN1 peptide content was differentially regulated by alcohol treatment in the two genotypes: it was significantly decreased in WT mice (*p* < 10^−3^) while it increased in *Trpa1* KO animals (*p* = .002) (Figures 5B, D).

### 3.6 Dynamics of CART mRNA and peptide upon alcohol treatment

To study the effect of alcohol on *Cart* mRNA and CART peptide content in the EWcp neurons, RNAscope ISH and immunofluorescence were performed, respectively.

**FIGURE 6:**
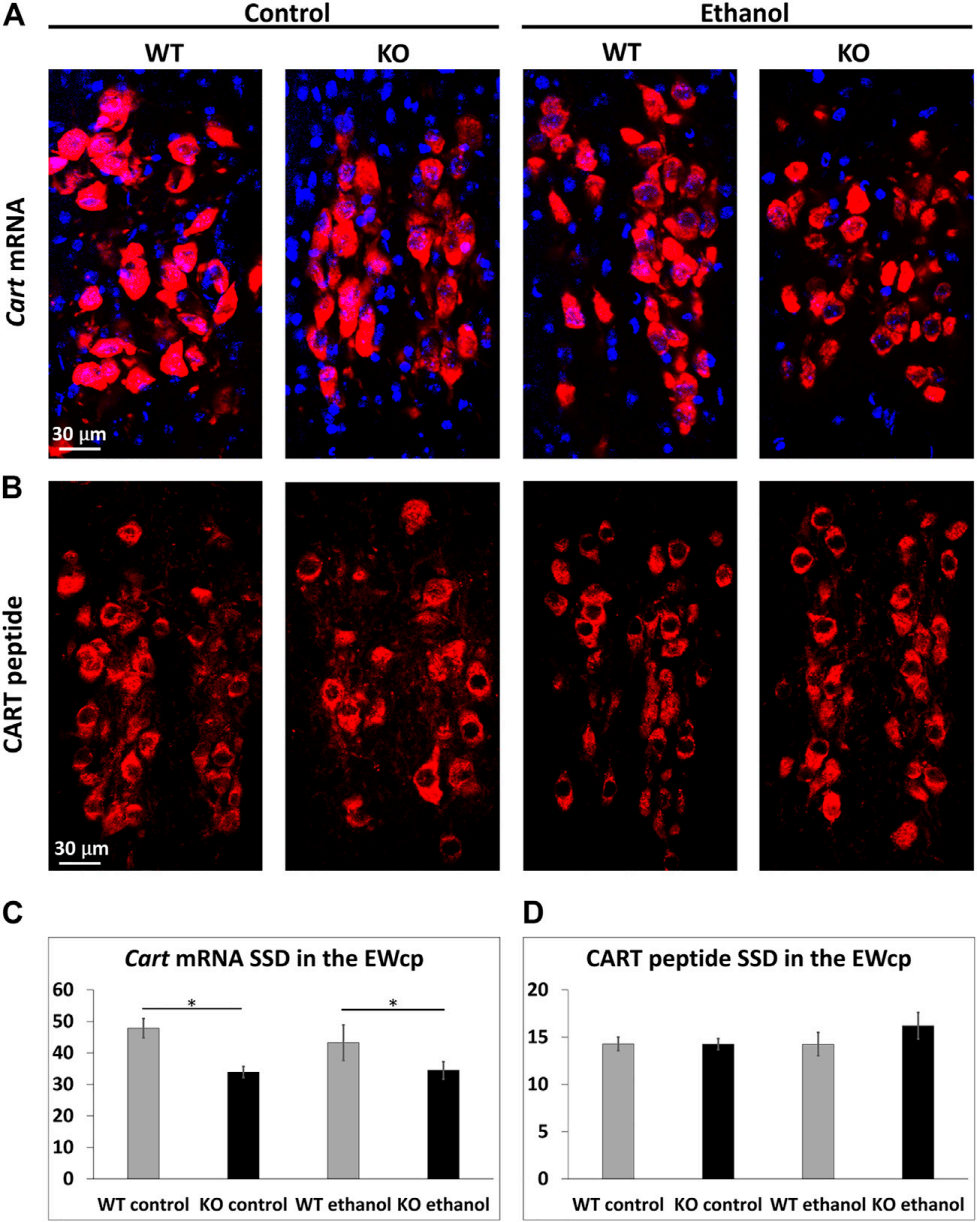
CART mRNA and peptide content of the centrally projecting Edinger–Westphal nucleus (EWcp). Representative fluorescence images showing the expression of cocaine- and amphetamine-regulated transcript (*Cart*) mRNA (red) by RNAscope *in situ* hybridization **(A)** and the CART peptide (red) by immunofluorescence **(B)**, in the EWcp of wildtype (WT) and *Trpa1* knockout (KO) mice 2 h after i.p., saline (control) and 1 g/kg ethanol injection. For nuclei, the sections were counterstained with 4′,6-diamidino-2-phenylindole (DAPI) (blue). Quantitative evaluation of *Cart* mRNA **(C)** and CART peptide **(D)** specific signal density (SSD) in the EWcp of WT and *Trpa1* KO mice, 2 h after i.p., saline (control) and 1 g/kg ethanol injection. Columns show means ± SEM of *Cart* mRNA **(C)** and CART peptide **(D)** SSD in the EWcp (*n* = 6–8; *p = .05; two-way ANOVA and Tukey’s *post hoc* test).

There was a main effect of the genotype (ANOVA: F_1,21_ = 10.37; *p* = .004) on *Cart* mRNA expression. In KO animals, a lower expression of *Cart* was observed regardless the treatment condition (Figures 6A, C).

The statistical evaluation found neither a main effect of alcohol treatment nor that of genotype on CART peptide content (Figures 6B, D).

## 4 Discussion

Our research group previously proved the presence of the *Trpa1* mRNA in mouse and human UCN1-immunoreactive neurons in the EWcp (Kormos et al., 2022). Here, we used an electrophysiological tool to prove the existence of the functionally active TRPA1 channel in the EWcp.

Presence of TRPA1 has been shown previously in cerebral blood vessels (Earley et al., 2009) and astrocytes (Shigetomi et al., 2012) of the mouse brain. However, the functional existence of TRPA1 in neurons of the brain was not shown before. Early studies suggested that pharmacological blockade of TRPA1 channel can be protective in granule cell degeneration (Koch et al., 2011) however this study did not prove the presence of the channel by histological experiments. Recently, we have shown using RNAscope *in situ* hybridization that *Trpa1* transcripts are present in urocortinergic neurons of the EWcp. Here, we suggest that TRPA1 is functionally active in these neurons. UCN1-immunoreactive neurons are spontaneously active and fire APs at resting membrane potential. Our hypothesis was that activation of TRPA1 in these neurons will result in Ca^2+^ influx and subsequent membrane potential depolarization which in turn will increase the frequency of spontaneous firing. Indeed, application of JT010, a selective and potent TRPA1 agonist, significantly increased the spontaneous firing frequency of UCN1-immunoreactive neurons while it was ineffective in neighboring neurons lacking UCN1. To our knowledge, this is the first evidence suggesting the functional role of TRPA1 in neurons of the mouse brain.

In our model for acute alcohol exposure, the measurement of urine alcohol concentration proved the reliability of the model as the absorption of ethanol was identical in WT and Trpa1 KO mice. Ethanol and all its metabolites efficiently pass through the blood-brain barrier (Nurmi et al., 1999; Quertemont and Tambour, 2004), and they were also shown to activate the TRPA1 *in vitro* (Bang et al., 2007; Wang et al., 2011; Komatsu et al., 2012) therefore, we propose that they may directly act on TRPA1 receptors in the EWcp. Increased FOS expression upon alcohol treatment further supports that alcohol could activate the EWcp urocortinergic neurons in WTs (Supplementary Figure S3), which is consistent with the literature (Bachtell et al., 1999; Ryabinin et al., 2001; Weitemier et al., 2001; Zuniga and Ryabinin, 2020). Our present finding, that the FOS activation was observed in alcohol-treated *Trpa1* KO mice also, suggests that besides the TRPA1 other receptors/ion channels may contribute to the alcohol-induced activation of urocortinergic cells. In line with this assumption, Bachtell et al. (2002) proposed that the alcohol-induced FOS response in EWcp is a result of signaling via GABA-A receptors, modified by *α*2A/D-adrenoceptors and dopamine receptors (Bachtell et al., 2002). Another possibility is that the alcohol-induced FOS activation in the EWcp is at least in part orchestrated through a TRPA1-independent mechanism by another alcohol-responsive brain area that innervates to the urocortinergic cells of the EWcp (Ryabinin et al., 1997; da Silva et al., 2013).

Because of the lack of genotype effect in the FOS cell counts, our data do not suggest unequivocally the role of TRPA1 in the EWcp, the reduced *Trpa1* mRNA expression in WT mice upon ethanol treatment, provides a further support for this assumption. Indeed, it is well-known that the effect of an agonist may downregulate its target (Finch et al., 2009). The fact that the *Trpa1* transcripts were restricted to the cells with UCN1 signal in both groups, on one hand replicated our recent finding that exclusively urocortinergic cells of the EWcp express the *Trpa1* (Kormos et al., 2022) on the other hand this indicates that acute alcohol treatment does not induce the transcription of *Trpa1* mRNA in non-urocortinergic EWcp cells, because we did not see any *Trpa1* mRNA transcripts outside the UCN1 neurons of the EWcp.

The UCN1 peptide content in EWcp differed between the two genotypes in saline-treated groups. The UCN1 content was much higher in WTs, compared to *Trpa1* KO mice. The comparison of the changes of UCN1 peptide content upon alcohol treatment revealed an opposite dynamics, with a decrease in WT mice, and an increase in *Trpa1* KO animals. This suggests that in WT mice the UCN1 is released from EWcp/UCN1 neurons in response to ethanol, while in *Trpa1* KO mice, increased UCN1 peptide content was observed suggesting the accumulation of the UCN1 peptide, possibly due to a reduced release. This was in part further supported by the RNAscope ISH, where we found lower *Ucn1* mRNA expression in the alcohol-treated *Trpa1* KO mice, compared to the WTs. This suggests that the peptide accumulation was associated with lower mRNA production due to the slower turnover (Gaszner et al., 2009).

Both control and alcohol-treated *Trpa1* KO animals showed lower *Cart* mRNA expression than the WT counterparts. Because the lower *Cart* mRNA expression is associated with reduced alcohol preference (Giardino et al., 2017), in our ongoing experiments we test if this is indeed characteristic for *Trpa1* KO mice. Neither the *Cart* mRNA expression, nor the CART peptide content of EWcp/UCN1 neurons was altered by acute alcohol treatment in any genotypes, suggesting that acute alcohol exposure does not have a deep impact on EWcp/CART. Based on the known role CART in addiction (Vicentic and Jones, 2007; Zuniga and Ryabinin 2020; Ong and McNally, 2020) we predict that, a chronic alcohol exposure model could prove its recruitment in alcohol abuse.

These above discussed observations together suggest that TRPA1 signaling may be involved in both the storage and release of UCN1 peptide from EWcp/UCN1 neurons. In our previous study we also found that the lack of functionally active TRPA1 affected the UCN1 content both in models of depression (Kormos et al., 2022) and posttraumatic stress disorder (Konkoly et al., 2022). In these models, we detected a genotype-related difference in the basal *Ucn1* mRNA (but not peptide) content in naïve control animals (Kormos et al., 2022; Konkoly et al., 2022). In contrast, in the present study saline-injected controls did not show a genotype difference in *Ucn1* mRNA expression but the peptide did. The discrepancy may be explained by the high acute stress sensitivity of the nucleus (Gaszner et al., 2004; Kormos et al., 2016) and by the stress effect of the ip., injection procedure.

The activation of TRPA1 in the membrane leads to calcium influx, triggering several intracellular pathways (Song and Guo, 2019). Indeed, our electrophysiological experiments showed that activation of TRPA1 increased the excitability and the rate of spontaneous firing of UCN1-expressing neurons leading to elevated calcium level. The increased intracellular calcium may cause exocytosis of the neuropeptide containing vesicles. Further experiments using pharmacological tools and electrophysiological recordings are required to the determine how exactly TRPA1 signaling contributes to the content and release of the UCN1 peptide. Considering the fact that lack of TRPA1 affected only UCN1 but not CART content of EWcp neurons regardless their co-localization (Kozicz, 2003; Priest et al., 2021; Li and Ryabinin 2022), which we also confirmed here (Supplementary Figure S4), it will be important to investigate the mechanism of UCN1-specific regulatory role of the TRPA1 channel.

## 5 Limitation

When assessing our present results, some limitations have to be considered. Because the functional TRPA1 receptor was deleted both in the periphery and the CNS in our global knockout mouse strain, we cannot exclude the possibility that peripheral or central compensatory mechanisms contributed to the alterations of the examined variables, observed in present study. We cannot exclude that possible litter differences influenced our results as WT and KO mice were not littermates. Because, at the moment, no reliable TRPA1 receptor antibody is available, we are unable to support our findings at protein level. The *in vivo* pharmacological manipulation on the TRPA1 receptor was also not possible due to the lack of information on the safety and pharmacokinetic profile of selective TRPA1 agonists and antagonists. We did not use alcohol for the electrophysiological experiment, because we assume, that the effect of the alcohol is not TRPA1 selective (Pozos and Oakes, 1987; Crews et al., 1996).

## 6 Conclusion and future perspective

With respect to the above listed limitations, in this study we proved the presence of functional TRPA1 receptors on UCN1 neurons of the EWcp. Decreased *Trpa1* mRNA expression upon acute alcohol treatment associated with reduced neuronal UCN1 peptide content suggests that this cation channel may contribute to the regulation of the UCN1 release. Taking the involvement of EWcp/UCN1 in chronic alcohol consumption and addiction in consideration (Schreiber and Gilpin, 2018; Zuniga and Ryabinin, 2020), in our ongoing research, we are investigating the recruitment of EWcp/TRPA1/UCN1/CART neurons in mouse models of chronic alcohol abuse.

## Data Availability

The raw data supporting the conclusion of this article will be made available by the authors, without undue reservation.
