# Supplementary material for: Functionally active TRPA1 ion channel is downregulated in peptidergic neurons of the Edinger-Westphal nucleus upon acute alcohol exposure
Source: Front Cell Dev Biol. 2023 Jan 10;10:1046559. doi: 10.3389/fcell.2022.1046559 (PMC9872022; doi:10.3389/fcell.2022.1046559)
Supplement: Supplementary file 1 [file DataSheet1.PDF]

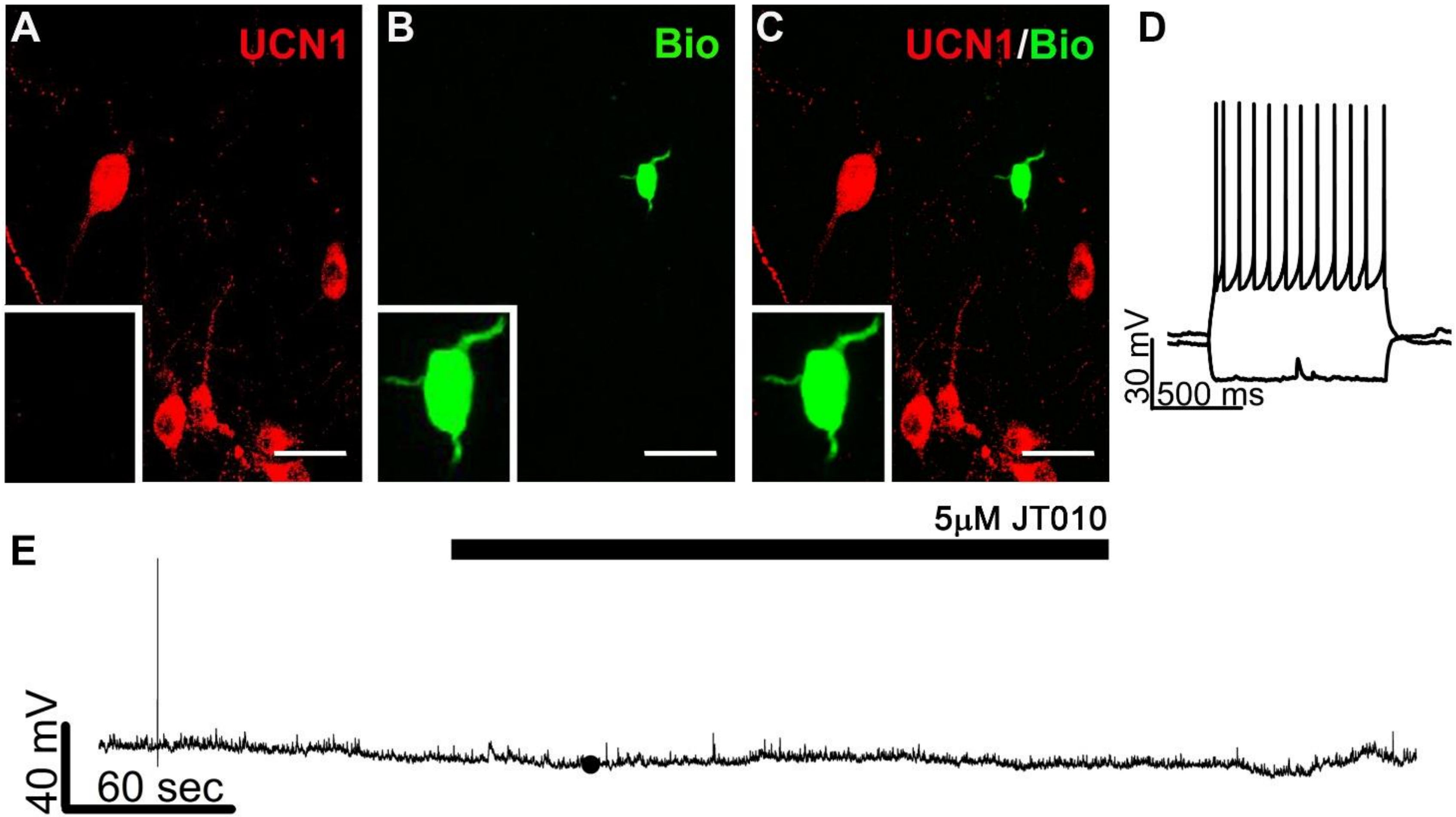

Supplementary Figure 1. JT010 does not alter spontaneous activity of UCN1-immunonegative neurons in the EW nucleus. Representative confocal images of UCN1 immunostaining (A, red), biocytin (B, green) filled patched neuron and the merged image (C) respectively. Insets show the magnified soma of the patched neuron. Note that the recorded neuron is UCN1-immunonegative. Scale bars: 40 μm. Respond of the recorded cell (D) to 1s current injection (-100 and +150 pA). Representative current clamp recordings (E) showing the spontaneous activity of the UCN1-immunnegative neuron. Black bar represents JT010 application (5 μM).

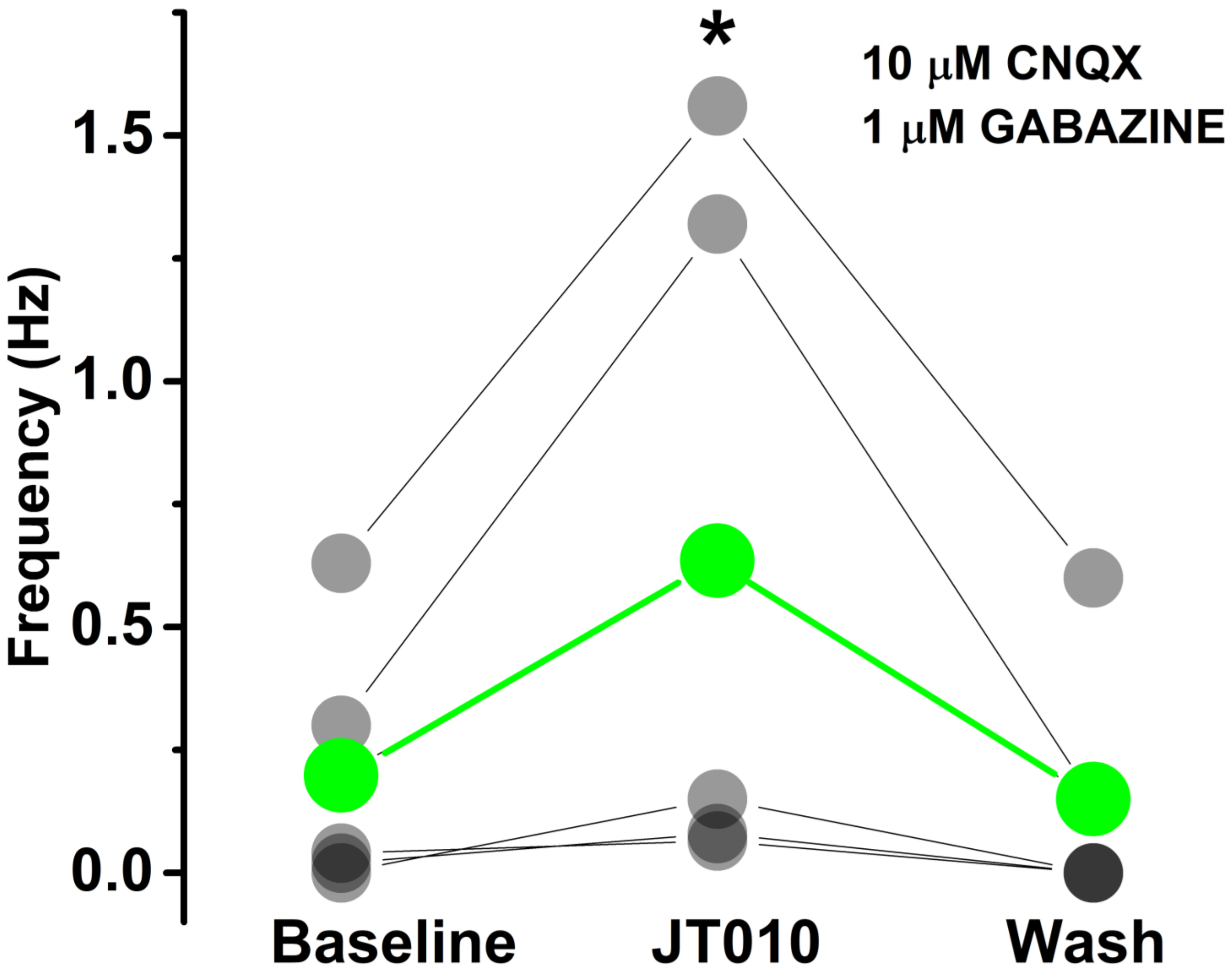

Supplementary Figure 2. Statistics showing the firing frequency at baseline (2 min before drug application), during JT010 application and after washing out the drug in the presence of CNQX and Gabazine (n=5 from 2 mice). *p<0.05; paired Student t test.

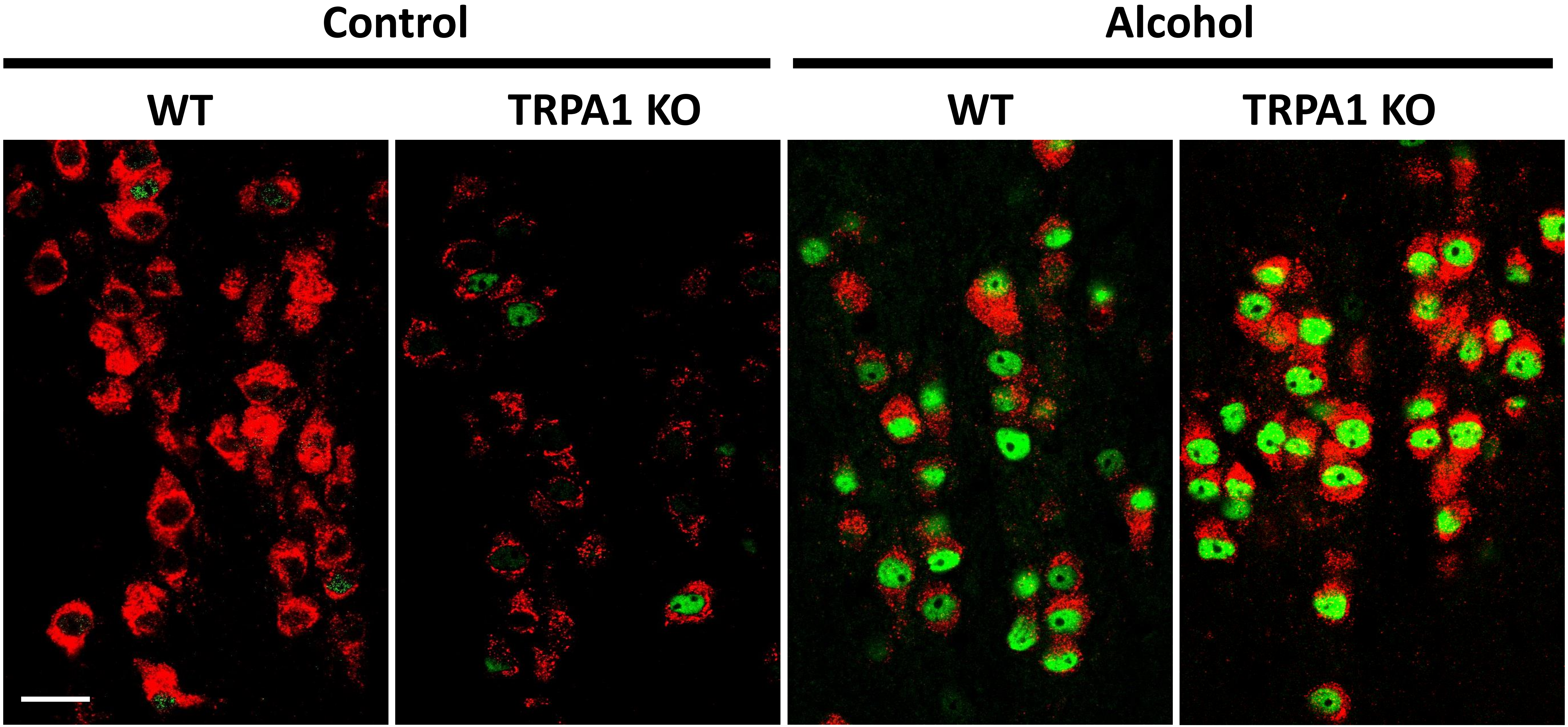

Supplementary Figure 3. Co-localization of urocortin1 (UCN1) and cFOS in the centrally projecting Edinger-Westphal nucleus (EWcp) neurons upon acute alcohol administration. Representative confocal images of the UCN1 (red) and cFOS (green) expression in the EWcp neurons by immunostaining. Note the colocalization of cFOS and UCN1. Scale bar: 20 μm.

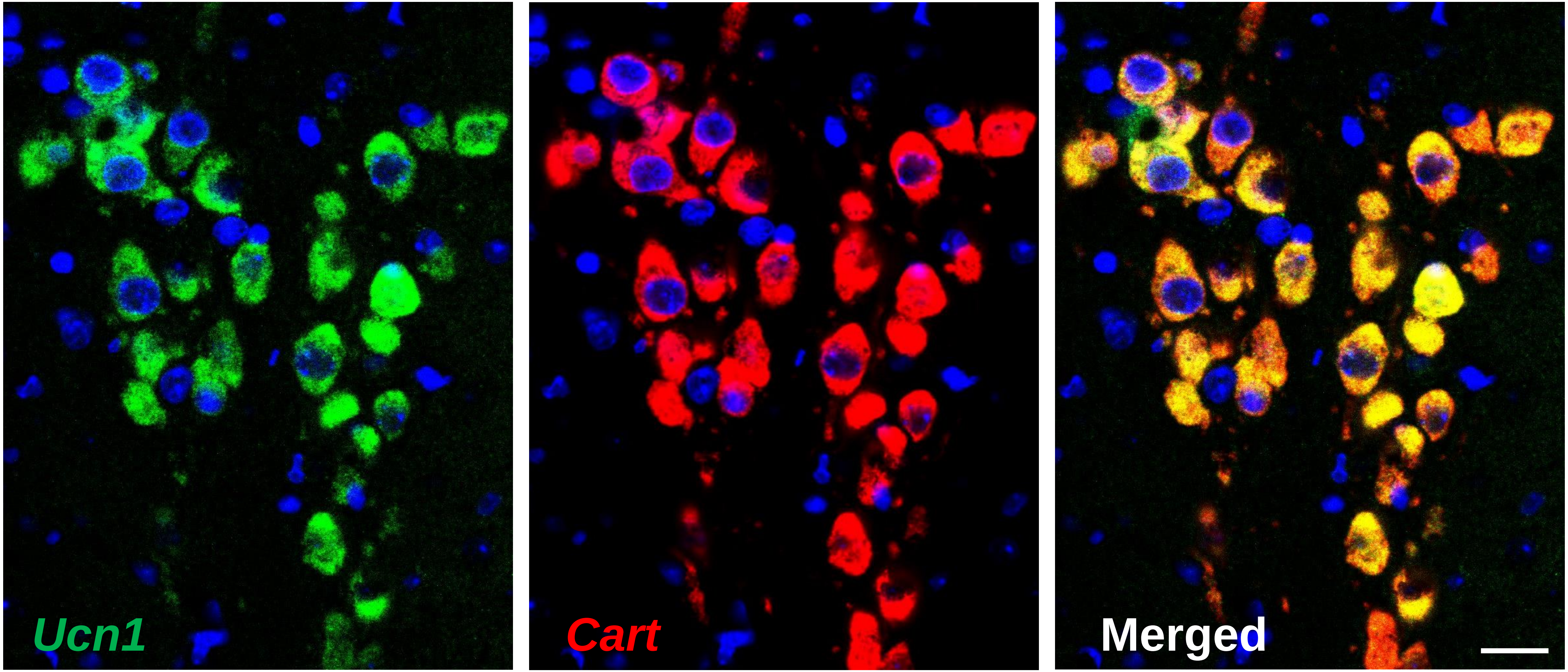

Supplementary Figure 4. Co-localization of urocortin1 (*Ucn1*) and cocaine and amphetamine-regulated transcript (*Cart*) in the centrally projecting Edinger-Westphal nucleus (EWcp) neurons. Representative confocal images of the *Ucn1* (green) and *Cart* (red) mRNA expression in the EWcp neurons by RNAscope *in situ* hybridization, counterstained with 4′,6-diamidino-2-phenylindole (DAPI; blue). Scale bar: 20 μm.
